# Perceived discrimination and psychological crisis among Chinese college students: a chain-mediation model of sense of life meaning and self-esteem

**DOI:** 10.3389/fpsyg.2025.1538653

**Published:** 2025-03-05

**Authors:** Lijuan Xu, Li Li

**Affiliations:** ^1^School of Education, Jiangxi Institute of Applied Science and Technology, Nanchang, China; ^2^Faculty of Psychology, Nanchang University, Nanchang, China

**Keywords:** perceived discrimination, psychological crisis, sense of life meaning, self-esteem, Chinese college student

## Abstract

**Background:**

Perceived discrimination constitutes an essential factor influencing the psychological crisis of college students. While prior research has examined the impact of discrimination on mental health in China, this study is the first to systematically investigate the chain mediating role of sense of life meaning and self-esteem in the relationship between perceived discrimination and psychological crisis. This approach not only enhances the theoretical framework of the relationship between discrimination and mental health but also offers a novel perspective for understanding discrimination-induced psychological crises in Chinese society.

**Objective:**

The present study used a questionnaire survey to test whether perceived discrimination may be associated with psychological crisis in Chinese college students. The mediating roles of sense of life meaning and self-esteem were also examined.

**Methods:**

In total, 514 college students were recruited to complete four scales, including the Perceived Discrimination Questionnaire, the Sense of Life Meaning Questionnaire, the Self-Esteem Scale, and the college student psychological crisis screening scale.

**Results:**

The findings are: (1) Perceived discrimination, sense of life meaning, and self-esteem have significant direct predictive effects on college students’ psychological crisis; and (2) sense of life meaning and self-esteem play a chain-mediating role in the relationship between perceived discrimination and psychological crisis of college students. The mediating effect includes two paths: perceived discrimination → self-esteem → psychological crisis (effect size: 0.130) and perceived discrimination → sense of life meaning → self-esteem → psychological crisis (effect size: 0.030).

**Conclusion:**

This research highlights that perceived discrimination can directly predict the psychological crisis of Chinese college students, and it can indirectly influence the level of psychological crisis of Chinese college students through the chain-mediating effect of sense of life meaning and self-esteem. The findings provide colleges and universities with valuable insights into the causes of students’ psychological crises, aiding in the adjustment of mental health education strategies and formulation of effective support systems for discriminated students. Additionally, this study offers a robust scientific foundation for policymakers to develop and promote anti-discrimination policies, and foster social harmony.

## 1 Introduction

Psychological crisis refers to the temporary psychological imbalance that occurs when an individual is unable of resolving the crisis events they face through existing coping strategies (Lemkau, 1964). It is manifested by changes in cognition, emotion, and behavior (Myer et al., 1992). The most common manifestation of emotional change is depression, and the most common manifestations of behavioral change are self-harm and suicide (Bian et al., 2010; Evans et al., 2004). College students constitute a high-risk group for psychological crisis, and the extremely negative events resulting from psychological crisis are increasing gradually. Studies indicate that the prevalence of depression among college students is 48.24%, suicide is becoming increasingly severe, and suicide mortality rate is increasing (Cui, 2021). Hence, it is important to explore the characteristics of Chinese college students’ psychological crisis and its mechanism of psychological development. Previous research has demonstrated that perceived discrimination (Han et al., 2020; Wang et al., 2020), sense of life meaning (Hui et al., 2020; Liu et al., 2023), self-esteem (Sun et al., 2024; Qi et al., 2024), and parenting style (Gao et al., 2023; Muris et al., 2004) might be factors influencing psychological crisis. Based on previous studies, this study investigates the factors associated with college students’ psychological crisis and the relationships between them.

### 1.1 Perceived discrimination and psychological crisis

The psychological mediation model holds that external stressful events or circumstances influence the emergence of psychological and behavioral issues by augmenting the negative attitudes internalized by individuals (Hatzenbuehler, 2009). The model encompasses two processes: First, the distal stress process, which pertains to exposure to stressful events that an individual is unable to cope with, such as bullying, discrimination, and rejection (Mereish et al., 2019). Second, the proximal process, which refers to the subjective vigilance of individuals with high levels of stressful events to the occurrence of events, manifests as an internalized negative attitude. That is, when processing external information, individuals believe that others will reject them, discriminate against them, and treat them unjustly. In previous investigations, the perception of discrimination constitutes an essential part of proximal processes (Staples et al., 2018). The perception of discrimination refers to an individual’s conviction that he or she is treated differently and unfairly by others within his or her group (Liu et al., 2013). It is a sort of universal subjective perception of exclusion, isolation, and prejudice, which can have a more direct impact on individual psychology and behavior than actual discrimination experiences (Dion and Kawakami, 1996). In a survey of female graduates from a university in North China who graduated in 2021, 68.6 percent reported experiencing employment discrimination. Specifically, 57.85 percent of the respondents encountered gender-based discrimination during their job search, exemplified by preferences for male candidates. Moreover, some students experienced severe incidents (such as depression, suicide, etc.) as a direct result of employment discrimination (Xia, 2022). Previous research has established a significant correlation between perceived discrimination and the psychological well-being of students (Song et al., 2021; Wang et al., 2020). Specifically, individuals with high levels of perceived discrimination have lower subjective happiness experiences, higher levels of depression and anxiety (Davis et al., 2016), and exhibit more suicide and self-harm behaviors (Delgado et al., 2017). The stress coping theory further postulates that when individuals discern discrimination, their own defense mechanism will be triggered by this danger indicator, causing the body to enter a state of stress and causing individuals to exhibit numerous stress responses over an extended period, such as depression, anxiety, and other mental health issues. Therefore, the current study proposes the following:

*H1*: Perceived discrimination has a significant positive predictive effect on Chinese college students' psychological crisis.

### 1.2 The mediating role of sense of life meaning

As a kind of positive psychological quality (Zheng, 2012; Yu and Chang, 2019), sense of life meaning exerts a positive influence on individuals’ stress resistance psychology (Zhang et al., 2021; Xu et al., 2020; Huang et al., 2020). Steger et al. (2008) proposed that life meaning pertains to individuals’ cognition and pursuit of their own life goals and divided life meaning into two dimensions: the possession of meaning and the pursuit of meaning. The greater meaning that an individual possesses, the more positive experiences they have. The loss recovery perspective (Li et al., 2018) contends that the search for life meaning predominantly occurs in individuals who have undergone setbacks. When confronted with adversity, individuals tend to pursue life meaning more, due to the reduction of meaning experience. Therefore, when individuals encounter stress, as a protective factor for mental health, a favorable outlook on life meaning will direct individuals toward a positive concept, which has a positive effect on individual depression (Lai and Xiao, 2018), health anxiety (Yek et al., 2017), and post-traumatic growth (Xiong et al., 2015). Furthermore, sense of life meaning is a protective factor that helps mitigate the risk of suicide (Bryan et al., 2019). Life meaning and reasons for survival serve as protective factors against suicidal ideation (Moscardini et al., 2021).The symbolic interaction theory (Charmaz et al., 2013) suggests that an individual’s self-concept is formed through the interaction between an individual and others. When college students perceive discrimination from the external world, they will transfer the prejudiced image onto themselves (Denison et al., 2020). After a prolonged period of internalization, they will become more in line with the prejudiced image and simultaneously adopt the method of escape to deal with it, which will ultimately diminish their sense of life meaning. Previous studies (Schmitt et al., 2014) have also discovered that the perception of discrimination will undermine the physical and mental health of individuals, and the impact on negative dependent variables (such as anxiety, depression, and negative emotions) is stronger than that on positive dependent variables (such as self-esteem, self-worth, and positive emotions), eventually leading to the conclusion that perceived discrimination will reduce sense of life meaning. Therefore, based on previous studies, this study considers sense of life meaning to be a mediating variable and proposes the following:

*H2*: Sense of life meaning plays a mediating role in the relationship between perceived discrimination and psychological crisis among Chinese college students.

### 1.3 The mediating role of self-esteem

In the study of psychological crisis, self-esteem is often examined as a mediating variable in relation to social support and its impact. Self-esteem refers to an individual’s enduring evaluation of themselves, reflecting their affirmation or negation of self-worth, as well as their beliefs regarding personal abilities, significance, success, and value. As an adaptive mechanism, self-esteem can provide protection for individuals when they encounter negative events and help them regulate adverse emotions (such as anxiety), thereby enabling them to lead a normal life (Leary et al., 1995). Furthermore, self-esteem is considered a core characteristic of mental health (Taylor and Brown, 1988). Research indicates that there is a close relationship between self-esteem and psychological crisis; higher levels of self-esteem can help college students to cope better with stressful life events, consequently reducing suicidal ideation and behaviors. Thus, it serves as a protective factor against psychological crisis (Li et al., 2024; Zhao and Wang, 2023). The social measurement theory of self-esteem (Zhang and Li, 2009) posits that individual self-esteem reflects the quality of interpersonal relationships; it symbolizes the connection between oneself and society or significant others. An individual’s objective experiences and subjective feelings significantly influence their level of self-esteem. When college students experience social stressors such as discrimination and rejection by others, they may utilize the adaptive mechanisms associated with self-esteem to assess their degree of acceptance within society and their interpersonal skills. They then adjust accordingly while integrating these assessments into their construction of self-cognition. Significantly, although short-term stressors do not necessarily lead to a decline in an individual’s level of self-esteem, prolonged exposure to stressors ultimately weakens one’s sense of esteem over time—thereby compromising physical and mental health. Empirical studies (Yang and Zhang, 2018; Fan et al., 2016) have also found that perceived discrimination negatively impacts adolescents’ self-esteem. Consequently, we propose the following:

*H3:* Self-esteem acts as an intermediary mechanism in the relationship between perceived discrimination and psychological crisis.

### 1.4 The chain-mediating role of sense of life meaning and self-esteem

Research indicates a significant positive correlation between sense of life meaning and self-esteem (Li et al., 2020; Liu and Wu, 2018). Wong’s theory of meaning management posits that an individual’s sense of meaning can regulate their self-perception, self-esteem, and self-evaluation, thereby facilitating a sense of purpose and fulfillment in life. This framework aids individuals in coping with various adversities, including the inevitability of death. Researchers (Gao et al., 2021; Wang, 2024) focusing on college students have identified that sense of life meaning serves as a predictor for self-esteem. Similarly, the Meaning Maintenance Model views self-esteem as an indicator for individuals to derive significance from their lives; thus, acquiring self-esteem is essential for sustaining one’s sense of life meaning. While investigating the predictive mechanisms by which self-esteem influences sense of life meaning, Chinese scholars (Liu and Wu, 2018) noted that factors such as psychological control sources play a role in this relationship. Both sense of life meaning and self-esteem are critical protective factors against psychological crisis; they may serve as buffers between perceived discrimination and psychological distress. Consequently, we hypothesize that perceived discrimination affects psychological crisis through the sequential mediation of sense of life meaning and self-esteem.

*H4*: There is a chain-mediation effect between sense of life meaning and self-esteem in the relationship between perceived discrimination and psychological crisis.

Based on the aforementioned background and assumptions, this study aims to explore the intrinsic mechanisms of perceived discrimination, sense of life meaning, self-esteem, and psychological crisis through the construction of a chain-mediation model. The goal is to reduce and prevent the occurrence of psychological crisis among college students. This research will provide a theoretical basis for schools and mental health service departments to implement prevention, early intervention, and management strategies for mental health issues faced by college students.

## 2 Materials and methods

### 2.1 Participants

A convenient sampling method was utilized to conduct a survey of Chinese college students in Jiangxi, Zhejiang, Guangdong, and Fujian provinces using an integration of online and paper questionnaires. The survey was conducted over a three-week period from April 8, 2024 to April 28, 2024. All participants participated voluntarily. Prior to the test, unified and standardized instructions were adopted to elucidate the answering approach and the investigation objective, and informed consent was given by the subjects. This study was sanctioned by the Academic Ethics and Ethics Committee of Nanchang University. A total of 548 questionnaires were distributed. After excluding invalid responses due to factors such as insufficient completion time or inconsistent reply patterns, 514 valid questionnaires remained, giving an effective response rate of 93.80%. Of the valid questionnaires, 68 were from first-year students (13.23%), 179 from second-year students (34.82%), 215 from third-year students (41.83%), and 52 from fourth-year students (10.12%). Also, 158 respondents (30.74%) identified as urban residents (from cities or provincial capitals), while 356 respondents (69.26%) came from rural areas (towns or villages). There were 43 only children among the participants (8.37%), and 471 with siblings (91.63%). Although convenience sampling may introduce selection bias, this study mitigates this potential limitation by collecting 514 valid data points, thereby forming a substantial sample size. This approach partially compensates for potential representativeness issues and enhances the reliability of the findings.

### 2.2 Measurements

#### 2.2.1 Perceived discrimination

The Perceived Discrimination Questionnaire, developed by Shen et al. (2009), consists of two dimensions: the Individual Perceived Discrimination Questionnaire and the Group Perceived Discrimination Questionnaire. The Individual Perceived Discrimination Questionnaire includes three items, while the Group Perceived Discrimination Questionnaire comprises three projects, utilizing a five-point Likert scale for scoring. A higher score indicates a greater level of discrimination perception among participants. This scale has demonstrated high reliability and validity through empirical research (Fang et al., 2008; Liu et al., 2013) and is widely applied in various studies. In this research, the internal consistency coefficient of this scale was found to be 0.896. Among these, 170 data points were selected to conduct a confirmatory factor analysis on the questionnaire, resulting in an excellent fit of the questionnaire structure (χ^2^/df = 2.560, CFI = 0.984, TLI = 0.971, and IFI = 0.985).

#### 2.2.2 Sense of life meaning

Sense of Life Meaning Questionnaire was developed by Steger et al. (2006) and is primarily designed to measure the perception and pursuit of life meaning of individuals. This questionnaire employs a 7-point Likert scale, where each item is scored from “1 = strongly disagree” to “7 = strongly agree.” A higher score indicates a stronger sense of life meaning. Wang X. et al. (2016) and Wang Y. et al. (2016) adapted this scale for use with university students, demonstrating good reliability and validity for assessing this population. In the present study, the reliability coefficient (*α*) of this scale was found to be 0.847, indicating that the Sense of Life Meaning Questionnaire possesses good reliability. Among these, 170 data points were selected to conduct a confirmatory factor analysis on the questionnaire, resulting in an excellent fit of the questionnaire structure (χ^2^/df = 2.211, CFI = 0.952, TLI = 0.937, and IFI = 0.953).

#### 2.2.3 Self-esteem

The Self-Esteem Scale (RSES), developed by Rosenberg in 1965, was employed to measure the self-esteem of middle school students (Rosenberg, 1965). The scale consists of 10 items. Scoring is based on a 4-point Likert scale, where 1 indicates “strongly agree” and 4 signifies “strongly disagree.” Items 3, 5, 9, and 10 are scored in the positive direction. Although the original scale includes five reverse-scored items, owing to cultural differences between Eastern and Western contexts, one item—“I wish I could earn more respect for myself”—has been adapted to be positively scored within the Chinese context (Tian and Li, 2005). In this study, the Cronbach’s alpha coefficient for RSES was found to be 0.866. Among these, 170 data points were selected to conduct a confirmatory factor analysis on the questionnaire, resulting in an excellent fit of the questionnaire structure (χ^2^/df = 3.242, CFI = 0.927, TLI = 0.903, and IFI = 0.928). The total score across all items represents an individual’s overall self-esteem level; higher scores indicate lower levels of self-esteem.

#### 2.2.4 Psychological crisis

The study utilized the college student psychological crisis screening scale developed by Chen (2019). This research employed a subscale for assessing psychological crisis among college students, which consists of 31 items across six dimensions: stress, suicidal ideation, interpersonal relationships, anxiety, self-worth, and family connection. The interpersonal relationships dimension includes six items, while each of the other dimensions contains five items. The scale employs a five-point Likert-type scoring system where “1” indicates never occurring and “5” indicates always occurring. This scale has been validated within the college student population, demonstrating a Cronbach’s *α* coefficient ranging from 0.805 to 0.941, indicating good reliability and validity (Chen, 2019). In this study, the Cronbach’s α coefficient for this scale was found to be 0.969. Among these, 170 data points were selected to conduct a confirmatory factor analysis on the questionnaire, resulting in an excellent fit of the questionnaire structure (χ^2^/df = 2.772, CFI = 0.893, TLI = 0.894, and IFI = 0.882).

### 2.3 Data analysis

The data were statistically analyzed using SPSS 27.0 and PROCESS 4.2. Common method bias was initially assessed using Harman’s single-factor analysis. Descriptive statistics were then employed, and the reliability of the scales was evaluated using Cronbach’s Alpha coefficient. Amos 26.0 was used for confirmatory factor analysis of the above four questionnaires. Pearson correlation coefficients were calculated to explore variable relationships. Finally, PROCESS (model 6) was utilized to investigate chain mediation relationships involving perceived discrimination, sense of life meaning, self-esteem, and psychological crisis.

## 3 Results

### 3.1 Results of the common method biases test

The reliance on self-reported data from the participants in this study introduces a potential risk of common method bias (Zhou and Long, 2004). To address this concern, we employed Harman’s single-factor test to examine all items related to the four variables, namely, perceived discrimination, sense of life meaning, self-esteem, and psychological crisis. The results indicated that there were nine factors with eigenvalues greater than 1. The first factor accounted for 36.926% of the variance explained, which is below the critical threshold of 40%. This suggests that there is no significant issue with common method bias in this study.

### 3.2 Descriptive statistics and correlation analyses of the research variables

The descriptive statistical results and correlation analysis for each variable in this study are presented in Table 1. The results indicate that all correlations between the variables reached significant levels, specifically: there is a significant positive correlation between perceived discrimination and psychological crisis (*r* = 0.619, *p* < 0.001); sense of life meaning was negatively correlated with perceived discrimination (*r* = −0.208, *p* < 0.001) and psychological crisis (*r* = −0.319, *p* < 0.001); and self-esteem scores are significantly positively correlated with perceived discrimination and psychological crisis (r_1_ = 0.471, *p* < 0.001; r_2_ = 0.617, *p* < 0.001). It is important to note that higher scores on the RSES indicate lower levels of self-esteem; therefore, self-esteem is negatively correlated with both perceived discrimination and psychological crisis. Additionally, sense of life meaning shows a significant negative correlation with self-esteem scores (*r* = −0.503, *p* < 0.001), which similarly implies a significant positive relationship between sense of life meaning and self-esteem when considering that higher RSES scores reflect lower self-esteem levels. The correlation between the variables provides a foundation for the validation of subsequent hypotheses.

**Table 1:** Descriptive statistics and correlation matrix of each variable (*n* = 514).

Variable	M ± SD	1	2	3	4	5	6	7	8
1. Gender	1.603 ± 0.489	–							
2. Grade	2.488 ± 0.848	−0.030	–						
3. Place of Origin	1.693 ± 0.462	0.089^*^	0.041	–					
4. Whether you are the only child	1.916 ± 0.277	0.143^***^	0.025	0.179^***^	–				
5. Perceived discrimination	2.266 ± 0.773	0.745^***^	0.015	0.177^***^	0.142^***^	–			
6. Sense of life meaning	4.669 ± 0.862	−0.146^***^	0.065	−0.123^***^	−0.107^*^	−0.208^***^	–		
7. Self-esteem	1.994 ± 0.424	0.338^***^	−0.041	0.143^***^	0.180^***^	0.471^***^	−0.503^***^	–	
8. Psychological crisis	2.298 ± 0.648	0.426^***^	−0.030	0.053	0.108^*^	0.619^***^	−0.319^***^	0.617^***^	–

^*^ *p* < 0.05, ^**^ *p* < 0.01, ^***^ *p* < 0.001.

### 3.3 Analysis of mediation effects

A multiple regression model was constructed with perceived discrimination, sense of life meaning, and self-esteem as independent variables, and psychological crisis as the dependent variable. The resulting variance inflation factor (VIF) values were all less than 5, indicating an absence of multicollinearity among the predictors. To further investigate the correlation between perceived discrimination, sense of life meaning, self-esteem, and psychological crisis, we employed Model 6 of the SPSS macro program PROCESS developed by Hayes to conduct multiple hierarchical regression analyses on the data. With perceived discrimination as the independent variable, the psychological crisis of college students as the dependent variable, and sense of life meaning and self-esteem as the mediating variables, Gender, Grade, Place of Origin, and Whether you are the only child as control variables, this paper examines the chain effect of sense of life meaning and self-esteem on the relationship between perceived discrimination and the psychological crisis of college students, and performs a regression analysis. The analysis results are presented in Table 2.

**Table 2:** Regression analysis of the research variables.

Variable	Sense of life meaning			Self-esteem			Psychological crisis		
	*β*	SE	*t*	*β*	SE	*t*	*β*	SE	*t*
Gender	0.045	0.114	0.392	−0.024	0.045	−0.537	−0.099	0.061	−1.625
Grade	0.075	0.044	1.719	−0.011	0.017	−0.663	−0.013	0.023	−0.563
Place of Origin	−0.149	0.083	−1.803	0.010	0.033	0.311	−0.122	0.044	−2.755
Whether you are the only child	−0.214	0.137	−1.558	0.127	0.054	2.345	−0.027	0.074	−0.369
Perceived discrimination	−0.227	0.073	−3.114^***^	0.215	0.029	7.427^***^	0.413	0.041	9.992^***^
Sense of life meaning				−0.204	0.017	−11.684^***^	−0.026	0.027	−0.959
Self-esteem							0.621	0.060	10.311^***^
*R* ^2^	0.061			0.401			0.529		
*F*	6.570^***^			56.669^***^			81.333^***^		

^*^ *p* < 0.05, ^**^ *p* < 0.01, ^***^ *p* < 0.001.

**Figure 1:**
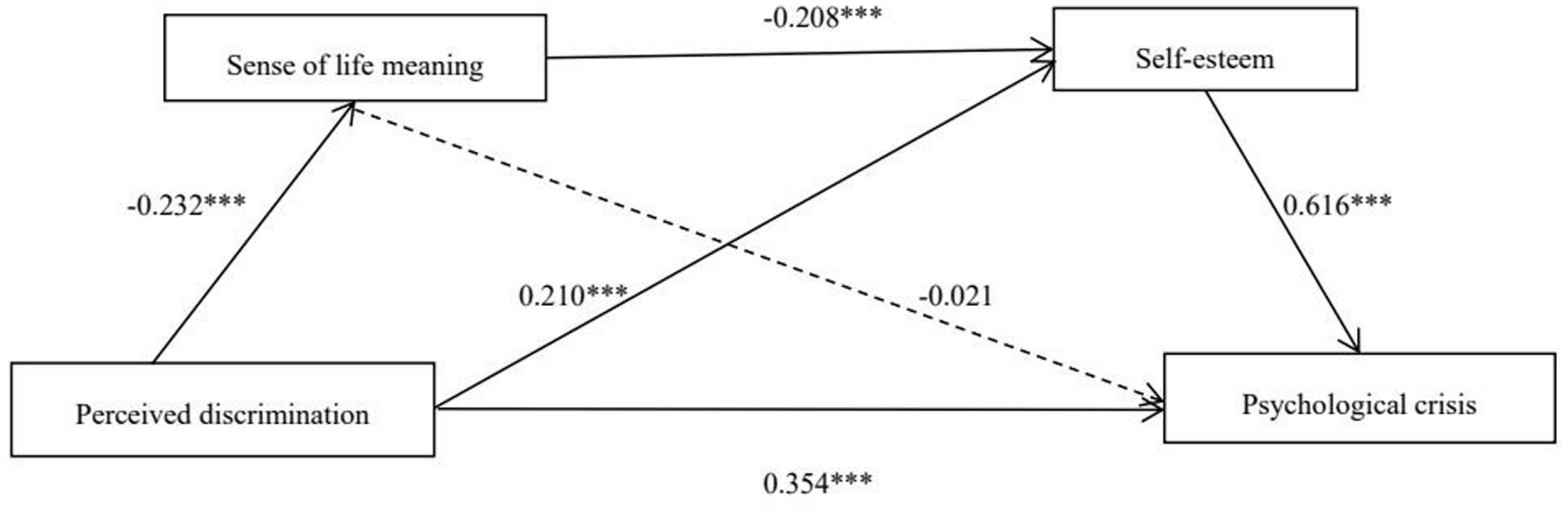
Serial mediation model (* *p* < 0.05, ** *p* < 0.01, *** *p* < 0.001).

The results indicated that perceived discrimination could not only negatively predict sense of life meaning (*β* = −0.227, *t* = −3.114, *p* < 0.001), but also positively predict the self-esteem score (*β* = 0.215, *t* = 7.427, *p* < 0.001). Nevertheless, as a higher score on the RSES indicates a lower level of self-esteem, perceived discrimination could negatively predict the level of self-esteem. Additionally, sense of life meaning could negatively predict the self-esteem score (*β* = −0.204, *t* = −11.684, *p* < 0.001), but given that a higher a score on the RSES indicates a lower level of self-esteem, sense of life meaning could positively predict the level of self-esteem. Finally, perceived discrimination and self-esteem score can positively predict the psychological crisis of college students (*β*_1_ = 0.413, *t* = 9.992, *p* < 0.001; *β*_2_ = 0.621, *t* = 10.311, *p* < 0.001), but because a higher score on the RSES indicates a lower level of self-esteem, self-esteem can negatively predict the level of the psychological crisis of college students (see Figure 1).

Subsequently, we utilized the bootstrap method to examine and develop the chained mediation model. The number of draws was established at 5000 and the confidence interval was set at 95% to assess the significance of the total, direct, and mediating effects. If none of the path coefficients include 0 within the 95% confidence interval, the statistic is considered statistically significant, signifying a significant mediation effect (Wen and Ye, 2014). The results of the path coefficient test are shown in Table 3. Table 3 presents the results indicating that the overall impact of perceived discrimination on psychological crisis is 0.518, with a corresponding 95% confidence interval of [0.461, 0.575]. The direct effect value of perceived discrimination on psychological crisis is 0.354, accounting for 68.340% of the total effect, and its associated 95% confidence interval is [0.297, 0.412], which does not include 0. Thus, the direct effect is significant and supports hypothesis H1; that is, perceived discrimination has a significant predictive effect on psychological crisis. The predictive effects of the three mediating paths on psychological crisis are as follows: Path 1: Perceived discrimination → sense of life meaning → psychological crisis has a corresponding 95% confidence interval of [−0.008, 0.021], which includes 0; therefore, the mediating effect of sense of life meaning in the relationship between perceived discrimination and psychological crisis is not significant, leading to the rejection of hypothesis H2. Path 2: Perceived discrimination → self-esteem → psychological crisis has a corresponding 95% confidence interval of [0.091, 0.173], which does not include 0; this indicates a significant mediating effect with an effect size proportioning at 25.097%. This suggests that perceived discrimination can predict psychological crisis through self-esteem, thereby supporting hypothesis H3. More formally, self-esteem acts as an intermediary mechanism in the relationship between perceived discrimination and psychological crisis. Path 3: Perceived discrimination → sense of life meaning → self-esteem → psychological crisis has a corresponding 95% confidence interval of [0.015, 0.048], which also does not include 0; this indicates an effect size proportioning at 5.792%. This finding confirms that sense of life meaning and self-esteem serve as chain mediators between perceived discrimination and psychological crisis, thus supporting hypothesis H4. In other words, there is a chain-mediation effect between sense of life meaning and self-esteem in the relationship between perceived discrimination and psychological crisis.

**Table 3:** The results of mediating effect test.

Model paths	Effect size	Boot SE	95% LLCI	95% ULCI	Ratio
Direct effect					
Perceived discrimination → psychological crisis	0.354	0.030	0.297	0.412	68.340%
Indirect effect					
Path 1: perceived discrimination → sense of life meaning → psychological crisis	0.004	0.007	−0.008	0.021	0.771%
Path 2: perceived discrimination → self-esteem → psychological crisis	0.130	0.021	0.091	0.173	25.097%
Path 3: perceived discrimination → sense of life meaning → self-esteem → psychological crisis	0.030	0.008	0.015	0.048	5.792%
Total effect	0.518	0.030	0.461	0.575	

LLCI, lower limit of the confidence interval; SE, standard error; ULCI, upper limit of the confidence interval.

## 4 Discussion

This study comprehensively examines the relationships between perceived discrimination, sense of life meaning, self-esteem, and psychological crisis among college students in China. The findings indicate that perceived discrimination can predict psychological crises by affecting self-esteem. Additionally, the interaction between sense of life meaning and self-esteem plays a mediating role in the development of psychological crisis. These insights provide a new perspective for understanding psychological crises in college students and offer a scientific basis for formulating effective prevention and intervention measures. The research underscores the importance of enhancing college students’ self-esteem and a sense of life meaning as crucial strategies for preventing psychological crises, which is significant for promoting their mental health and overall development.

### 4.1 Direct prediction of perceived discrimination on college students’ psychological crisis

This study has found a close relationship between perceived discrimination and the level of psychological crisis among college students, thereby validating hypothesis H1. This finding is consistent with previous research results. For instance, perceived discrimination significantly positively predicts individual depression (Brody et al., 2006) and self-harm as well as suicide (Sutin et al., 2018). According to the Rejection-Identification Model (RIM) (Li and Lu, 2018), perceived discrimination may lead individuals to feel rejected by external groups, making them aware of their own group’s disadvantaged position and vulnerability, which in turn adversely affects their mental health. This phenomenon can be attributed to two main factors: First, high levels of discrimination may cause individuals to become more sensitive to others’ opinions during peer interactions and perceive others’ attitudes as discriminatory. To mitigate the negative experiences associated with this perception of discrimination, individuals might actively avoid interpersonal interactions, resulting in a certain degree of impairment in their social skills (Myrick and Martorell, 2011). Second, individuals with high levels of perceived discrimination tend to exhibit lower levels of self-disclosure (Ragins et al., 2007). When negative events occur without adequate listeners or peer support available to them, they are more likely to develop emotional and behavioral disorders. Thus, it is evident that the perception of discrimination is a significant factor influencing psychological crises among university students. To mitigate the impact of discriminatory perceptions on these crises, schools should aim to foster an equitable and inclusive campus environment through anti-discrimination education, comprehensive support systems, and a culture of inclusivity. First, anti-discrimination education is essential. Integrating courses and activities that enhance students’ awareness and understanding of discrimination can cultivate empathy and promote inclusiveness. This approach not only mitigates discriminatory behavior but also fosters a harmonious campus atmosphere. Empirical evidence suggests that such educational initiatives significantly reduce incidents of bias and prejudice, thereby enhancing the overall culture on campus. Second, establishing a robust support system is crucial. Providing psychological support and counseling services to students who have experienced discrimination helps them manage negative emotions and psychological stress. Professional psychological assistance offers a safe space for students to express themselves, aiding in the restoration of their confidence and mental well-being. Studies have shown that access to these resources can lead to improved academic performance and personal resilience among affected students. Third, creating an inclusive campus culture requires ongoing efforts. Diversity and inclusion training for faculty and staff ensures that the campus environment remains safe and welcoming for all students. This initiative necessitates collaboration from management to frontline personnel, fostering an environment where every student feels respected and accepted. Continuous evaluation and adaptation of these training programs are essential to address emerging challenges and maintain an inclusive atmosphere. Implementing these measures will contribute significantly to building a more equitable and inclusive campus, thereby ensuring the healthy physical and mental development of students. Research indicates that such environments positively impact student retention and overall satisfaction with their educational experience.

### 4.2 Indirect prediction of perceived discrimination on college students’ psychological crisis

#### 4.2.1 Mediating role of sense of life meaning

This study delves into the mediating function of sense of life meaning within perceived discrimination and psychological crisis, and the results reveal that the mediating impact is negligible. Hypothesis H2 remains unsubstantiated. Through dissecting the two halves of the mediated path, it was found that in the first half of the path, perceived discrimination exerts a remarkable predictive influence on sense of life meaning. This effect aligns with the outputs of previous empirical studies (Bai et al., 2022), specifically that perceived discrimination can reduce an individual’s sense of life meaning.

In the last section of the mediating path, sense of life meaning fails to exert a significant predictive influence on psychological crisis among college students. These results are at variance with the theoretical expectations of this study, indicating that individuals with a low sense of life meaning do not necessarily possess a high level of psychological crisis. Several researchers posit that social support constitutes a critical environmental factor that significantly influences individual mental health (Wang et al., 2024). Social support not only buffers against the adverse effects of stress but also plays a pivotal role in maintaining positive emotional states (Chen and Li, 2015). When individuals experience social rejection, they tend to focus more on immediate concerns, thereby reducing their capacity for delayed gratification and potentially diminishing their sense of life meaning (Twenge et al., 2003). Conversely, adequate social support from family, friends, or social institutions can effectively mitigate psychological crises. Prior research has corroborated that the predictive impact of sense of life meaning on mental health is significantly influenced by the degree of social support, which plays a crucial role in this relationship (Miao et al., 2018; Wei et al., 2021). Several empirical studies have demonstrated that positive emotions can mitigate the impact of psychological crises to a significant extent (Ren, 2020). Even in cases where the sense of life meaning is diminished, individuals who experience higher levels of positive emotions such as happiness, satisfaction, and hope may be better able to maintain a healthy psychological state (Wang X. et al., 2016; Wang Y. et al., 2016). The connection between sense of life meaning and psychological crisis is not stable and might be influenced by the mediating or moderating effects of other potential variables (Wu and Li, 2022). Furthermore, interviews indicate that individuals with a low sense of life meaning do not necessarily experience a high level of psychological crisis.

These findings imply that the relationship between perceived discrimination and psychological crisis is not a single path but a complex process through which multiple psychological mediators interweave. It also suggests that multifaceted strategies should be adopted to prevent and intervene in the psychological crisis of college students, including enhancing individual self-esteem and belonging, strengthening social support and positive psychological resources, and improving the campus culture and environment to reduce the occurrence of discrimination and prejudice.

#### 4.2.2 The role of self-esteem as a mediator

In the analysis of mediating effects, we found that self-esteem plays a mediating role in the relationship between perceived discrimination and psychological crisis. This indicates that although perceived discrimination has an unavoidable direct impact on college students’ psychological crises, self-esteem can alleviate these crises. The research hypothesis H3 is thus validated, consistent with previous studies (Fan et al., 2016; Sowislo and Orth, 2013; Corning, 2002; Zuo et al., 2016). The interpersonal relationship theory of self-esteem suggests a close connection between self-esteem and self-cognition. When individuals face discriminatory environments or events, they may experience feelings of discrimination and shame. Individuals with high self-esteem typically possess positive self-perceptions and evaluations, which prevent them from internalizing adverse information as psychological issues. Conversely, those with low self-esteem are more likely to process negative information when confronted with discrimination, leading to increased levels of psychological crisis (Zuo et al., 2016). Furthermore, individuals with low self-esteem may experience heightened uncertainty regarding their own identity (Campbell, 1990). They often focus on negative portrayals related to themselves or their groups; this tendency is particularly pronounced among marginalized groups who are more susceptible to negative information processing, resulting in diminished personal and collective esteem. In social integration contexts, college students sometimes encounter discrimination or social exclusion; the level of their self-esteem significantly influences their psychological crisis levels. Individuals with high self-esteem can mitigate the negative impacts of discrimination by reinforcing group identity. In contrast, those with low self-esteem tend to have weaker coping mechanisms for adverse events and may experience psychological crises due to marginalization stemming from discriminatory experiences (Nie, 2016). Therefore, within the context of school activities, teachers should actively engage with students and provide constructive and positive feedback. Schools can enhance students’ self-esteem through the following three strategies: First, by implementing positive psychology courses and activities that assist students in identifying their unique strengths, thereby fostering self-esteem and confidence. Second, by encouraging students to set realistic goals and achieve a sense of accomplishment through goal attainment, thus effectively boosting self-esteem. Finally, by promoting collaborative group work and peer support to strengthen students’ sense of belonging and self-esteem. Through enhanced self-esteem, individuals can improve their sense of self-worth and self-efficacy, thereby reducing the risk of psychological distress.

#### 4.2.3 Chain mediation of sense of life meaning and self-esteem

This study also indicates that sense of life meaning and self-esteem play a chain mediating role between perceived discrimination and psychological crisis among college students. Specifically, perceived discrimination can first affect self-esteem through sense of life meaning, which subsequently influences psychological crisis in college students, thereby validating research hypothesis H4. The findings (Li, 2019) suggest that although individual differences—such as personal growth and life experiences—affect the level of perceived discrimination, high levels of perceived discrimination lead to a gradual decline in self-worth and happiness. This results in an increase in negative emotions such as anxiety, guilt, and shame, which naturally diminishes individuals’ sense of life meaning. According to terror management theory (Ryan and Deci, 2004), meaning and its perception contribute to self-esteem; they serve to defend against death awareness by overcoming fears associated with mortality and alleviating anxiety. When faced with threats, individuals tend to seek meaning through acceptance of surrounding cultural worldviews; this process enhances their sense of meaning while bolstering self-esteem and mitigating anxiety. Empirical studies have also shown that a higher sense of life meaning positively predicts self-esteem (Cao et al., 2019)—that is, individuals with a greater sense of purpose tend to exhibit higher levels of self-esteem in real-life contexts. This may be attributed to the fact that a strong sense of life meaning serves as an essential psychological resource (Liu and Wu, 2018); college students who possess a heightened sense of purpose are more likely to hold positive perceptions about themselves and exhibit confidence in their abilities. They maintain an optimistic outlook on life’s challenges due to their positive self-concept. Moreover, these students generally perceive social relationships more positively; they firmly believe in their own value and tend to be kind and trust in human goodness. Consequently, they manage interpersonal relationships more effectively and gain recognition from those around them, resulting in relatively high levels of self-esteem. Self-esteem not only reflects indicators related to psychological crisis but also constitutes significant influencing factors of such crises. Previous research has indicated that when students’ self-esteem is compromised, it may lead them toward severe psychological issues (Ma and Jiang, 2002; Jiang et al., 2024). Therefore, educational institutions should systematically implement curricula and activities centered on the exploration of sense of life meaning to assist students in understanding this profound topic. Concurrently, schools should offer timely psychological support services, including formal counseling and interventions, for students who exhibit a diminished sense of life meaning. Enhancing sense of life meaning can bolster their self-esteem, which is instrumental in fostering a healthy personality, improving mental well-being, and mitigating psychological crises among college students.

### 4.3 Research implications and limitations

This study focuses on the psychological crises experienced by college students, exploring the relationship between perceived discrimination and these crises, as well as the underlying mechanisms involved. The findings indicate that perceived discrimination is a significant predictor of psychological crisis among college students. Furthermore, self-esteem plays a mediating role in this process, while sense of life meaning and self-esteem jointly function as chain mediators. This research contributes to a deeper understanding of how perceived discrimination influences individual psychological crises through psychological mechanisms, providing a theoretical basis for designing effective mental health interventions. Additionally, it encourages higher education institutions and mental health professionals to develop targeted support programs aimed at enhancing college students’ sense of life meaning and levels of self-esteem, thereby equipping them to better cope with experiences of discrimination and associated psychological challenges.

This study has some limitations. First, in terms of research design, this study employs a cross-sectional approach, which precludes the exploration of causal relationships between the aforementioned variables. Future studies should employ longitudinal research designs to systematically investigate the dynamic relationships among the aforementioned variables over time. For instance, methods such as cross-lagged panel models (CLPM), multilevel linear models (MLM), and latent growth models (LGM) can be used to effectively analyze the temporal dynamics between psychological variables and psychological crises. Moreover, integrating qualitative methods can enhance the depth and richness of research. While quantitative approaches provide valuable insights into patterns and trends, qualitative methods, such as in-depth interviews and focus group discussions, offer detailed contextual information and nuanced narratives. Combining these methodologies can facilitate a more comprehensive understanding of the complex psychological mechanisms underlying the data. Second, this study only investigates the mediating roles of sense of life meaning and self-esteem in the relationship between perceived discrimination and psychological crisis among college students. It does not address other potential variables that may influence this relationship. Future studies could integrate multiple variables with perceived discrimination and psychological crisis to explore their interrelations further and identify additional mediating or moderating mechanisms at play.

## 5 Conclusion

To conclude, this study investigates the correlations between perceived discrimination, sense of life meaning, self-esteem, and psychological crisis. First, there are significant correlations between perceived discrimination, sense of life meaning, self-esteem, and psychological crisis. Second, self-esteem serves as a mediating factor between perceived discrimination and psychological crisis. Third, sense of life meaning and self-esteem function as chain mediators between perceived discrimination and psychological crisis. The findings of this study substantially advance the theoretical understanding of the relationship between perceived discrimination and psychological crisis. The results provide a robust foundation for the development of targeted intervention strategies to enable schools and society to guide students in appropriately perceiving and responding to discrimination through thoughtfully designed curricula, optimizing the school environment, and implementing a range of targeted measures. These initiatives will enhance psychological resilience and reinforce sense of life meaning and self-esteem, effectively reducing the incidence of psychological crises, safeguarding students’ mental health, and promoting their holistic development.

## Data Availability

The original contributions presented in the study are included in the article/Supplementary material, further inquiries can be directed to the corresponding author.
