# Supplementary material for: Perceived discrimination and psychological crisis among Chinese college students: a chain-mediation model of sense of life meaning and self-esteem
Source: Front Psychol. 2025 Mar 5;16:1538653. doi: 10.3389/fpsyg.2025.1538653 (PMC11920802; doi:10.3389/fpsyg.2025.1538653)
Supplement: Supplementary file 2 [file Table_2.docx]

Supplementary Material

Perceived Discrimination and Psychological Crisis among Chinese College Students:A Chain-mediation Model of Sense of Life Meaning and Self-esteem

Lijuan Xu^1^, Li Li^2*^

*** Correspondence:** Li Li: [lancylili@ncu.edu.cn](mailto:lancylili@ncu.edu.cn)

**1 Introduction**

Dear classmates, please help me with my academic research by completing this questionnaire. Your answers will provide me with valuable research data. This questionnaire is anonymous, and there is no right or wrong answer—instead, please choose the ones that best suit you. Thank you for participating in this survey!

The questionnaire consists of four parts composed of 6, 10, 10, and 31 questions, respectively. The questionnaire should take 5 min to complete. Thank you again for your attention.

**2 Basic Information**

1. Your grade: ① Freshman ② Sophomore ③ Junior ④ Senior

2. Originating Place: ① Urban (county, city, province) ② Rural (township, town, village)

3. Whether you are the only child: ① yes ② no.

**3 Perceived Discrimination Questionnaire ( D )**

Please read the following items carefully and select the one that best aligns with your preferences.

**d1** 4. I feel that I have been treated unfairly.

① Completely disagree ② Disagree somewhat ③ Unsure ④ Agree somewhat ⑤ Completely agree

**d2** 5. I believe that others look down on me.

① Completely disagree ② Disagree somewhat ③ Unsure ④ Agree somewhat ⑤ Completely agree

**d3**  6. Compared to others, I feel that I have lost certain opportunities.

① Completely disagree ② Disagree somewhat ③ Unsure ④ Agree somewhat ⑤ Completely agree

**d4** 7. Overall, students from similar family backgrounds as mine have been treated unfairly.

① Completely disagree ② Disagree somewhat ③ Unsure ④ Agree somewhat ⑤ Completely agree

**d5** 8. Overall, students from similar family backgrounds as mine have lost many opportunities.

① Completely disagree ② Disagree somewhat ③ Unsure ④ Agree somewhat ⑤ Completely agree

**d6** 9. Overall, students from similar family backgrounds as mine are looked down upon by others.

① Completely disagree ② Disagree somewhat ③ Unsure  ④ Agree somewhat ⑤ Completely agree

**4 Self-Esteem Scale ( E )**

The purpose of this response is to articulate your genuine self-perception, rather than what you believe you ought to convey. Please describe yourself in accordance with your usual and authentic circumstances.

**e1**  10. I feel that I am a valuable person, at least on par with others.

① Strongly agree ② Somewhat agree ③ Disagree ④ Strongly disagree

**e2**  11. I believe I possess many positive qualities.

① Strongly agree ② Somewhat agree ③ Disagree ④ Strongly disagree

**e3**  12. Ultimately, I tend to view myself as a failure.

① Strongly agree ② Somewhat agree ③ Disagree ④ Strongly disagree

**e4**  13. I can perform tasks as well as most people do.

① Strongly agree ② Somewhat agree ③ Disagree ④ Strongly disagree

**e5**  14. I feel there are not many aspects of myself that I'm proud of.

① Strongly agree ② Somewhat agree ③ Disagree ④ Strongly disagree

**e6**  15. I maintain a positive attitude toward myself.

① Strongly agree  ② Somewhat agree ③ Disagree ④ Strongly disagree

**e7**  16. Overall, I am satisfied with who I am.

① Strongly agree  ② Somewhat agree ③ Disagree  ④ Strongly disagree

**e8**  17. I wish to earn more respect for myself.

① Strongly agree  ② Somewhat agree ③ Disagree ④ Strongly disagree

**e9** 18. There are times when I truly feel useless.

① Strongly agree  ② Somehow agree   ③ Disagree   ④ Strongly disagree

**e10**  19. I often think that I'm worthless

① Strongly agree   ② Somehow agree  ③ Disagree   ④ Strongly disagree

**5 Sense of Life Meaning Questionnaire ( F )**

Guidance: Below are several sentences regarding the meaning of life and existence. Please rate how well these statements resonate with your personal experience using the following scale: 1 indicates "not at all applicable," 2 indicates "generally not applicable," 3 indicates "somewhat not applicable," 4 indicates "uncertain," 5 indicates "somewhat applicable," 6 indicates "generally applicable," and 7 indicates "completely applicable." Kindly mark a “√” next to the number that best reflects your situation.

**f1**  20. I am in search of a purpose or mission for my life.

① Completely disagree ② Mostly disagree ③ Somewhat disagree ④ Uncertain

⑤ Somewhat agree ⑥ Mostly agree ⑦ Completely agree

**f2** 21. My life lacks a clear purpose.

① Completely disagree ② Mostly disagree ③ Somewhat disagree ④ Uncertain

⑤ Somewhat agree ⑥ Mostly agree ⑦ Completely agree

**f3**  22. I am seeking the meaning of my life.

① Completely disagree ② Mostly disagree ③ Somewhat disagree ④ Uncertain

⑤ Somewhat agree ⑥ Mostly agree ⑦ Completely agree

**f4**  23. I understand the meaning of my life.

① Completely disagree ② Mostly disagree ③ Somewhat disagree ④ Uncertain

⑤ Somewhat agree ⑥ Mostly agree ⑦ Completely agree

**f5**  24. I am searching for things that make me feel my life is meaningful.

① Completely disagree ② Mostly disagree ③ Somewhat disagree ④ Uncertain

⑤ Somewhat agree ⑥ Mostly agree ⑦ Completely agree

**f6** 25. I constantly try to find the purpose of my life.

① Completely disagree ② Mostly disagree ③ Somewhat disagree ④ Uncertain

⑤ Somewhat agree ⑥ Mostly agree ⑦ Completely agree

**f7**  26. My Life Has A Clear Direction.

① Completely disagree ② Mostly disagree ③ Somewhat disagree ④ Uncertain

⑤ Somewhat agree ⑥ Mostly agree ⑦ Completely agree

**f8**  27. I Know What Can Make My Life Meaningful.

① Completely disagree ② Mostly disagree ③ Somewhat disagree ④ Uncertain

⑤ Somewhat agree ⑥ Mostly agree ⑦ Completely agree

**f9**  28. I Have Found A Satisfying Purpose In Life.

① Completely disagree ② Mostly disagree ③ Somewhat disagree ④ Uncertain

⑤ Somewhat agree ⑥ Mostly agree ⑦ Completely agree

**f10** 29. I Am Always Looking For Something That Makes My Life Feel Important.

① Completely disagree ② Mostly disagree ③ Somewhat disagree ④ Uncertain

⑤ Somewhat agree ⑥ Mostly agree ⑦ Completely agree

**6 psychological crisis screening scale ( G )**

Please read each of the following statements carefully and mark “√” in the option that best reflects your situation over the past month. The scale is as follows: 1 represents "never occurred"; 2 indicates "rarely occurred"; 3 signifies "occasionally occurred"; 4 denotes "frequently occurred"; and 5 means "always occurred."

**g1**  30. I feel that most people around me do not like me.

① Never occurs ② Rarely occurs ③ Occasionally occurs ④ Frequently occurs ⑤ Always occurs

**g2**  31. I wish to leave this world.

① Never occurs ② Rarely occurs ③ Occasionally occurs ④ Frequently occurs ⑤ Always occurs

**g3** 32. I often worry that things will spiral out of control.

① Never occurs ② Rarely occurs ③ Occasionally occurs ④ Frequently occurs ⑤ Always occurs

**g4** 33. I find it difficult to efficiently prepare for academic requirements at school.

① Never occurs ② Rarely occurs ③ Occasionally Occurs ④ Frequently Occurs ⑤ Always Occurs

**g5** 34. I feel inferior to others.

① Never occurs ② Rarely occurs ③ Occasionally occurs ④ Frequently occurs ⑤ Always occurs

**g6** 35. When facing difficulties, my family cannot provide help or support for me.

① Never occurs ② Rarely occurs ③ Occasionally occurs ④ Frequently occurs ⑤ Always occurs

**g7**  36. I struggle to integrate into my classmates' social groups.

① Never occurs ② Rarely occurs ③ Occasionally occurs ④ Frequently occurs ⑤ Always occurs

**g8**  37. Involuntary thoughts related to death frequently arise in my mind.

① Never occurs ② Rarely occurs ③ Occasionally occurs ④ Frequently occurs ⑤ Always occurs

**g9** 38. I often worry that bad things will happen to me.

① Never occurs ② Rarely occurs ③ Occasionally occurs ④ Frequently occurs ⑤ Always occurs

**g10**  39. I feel unable to manage my time effectively.

① Never occurs ② Rarely occurs ③ Occasionally occurs ④ Frequently occurs ⑤ Always occurs

**g11** 40. I feel that my wishes are almost impossible to fulfill.

① Never occurs ② Rarely occurs ③ Occasionally occurs ④ Frequently occurs ⑤ Always occurs

**g12** 41. I often find communication with my parents to be quite challenging.

① Never occurs ② Rarely occurs ③ Occasionally occurs ④ Frequently occurs ⑤ Always occurs

**g13**  42. I experience significant difficulties in social interactions.

① Never occurs ② Rarely occurs ③ Occasionally occurs ④ Frequently occurs ⑤ Always occurs

**g14**  43. I have contemplated suicide and considered methods for carrying it out.

① Never occurs ② Rarely occurs ③ Occasionally occurs ④ Frequently occurs ⑤ Always occurs

**g15**  44. I find it difficult to control feelings of worry or anxiety.

① Never occurs ② Rarely occurs ③ Occasionally occurs ④ Frequently occurs ⑤ Always occurs

**g16** 45. I am uncertain about how to plan my college life effectively.

① Never occurs ② Rarely occurs ③ Occasionally occurs ④ Frequently occurs ⑤ Always occurs

**g17**  46. I feel inadequate in everything I do.

① Never occurs ② Rarely occurs ③ Occasionally occurs ④ Frequently occurs ⑤ Always occurs

**g18** 47. During setbacks, my family is unable to provide me with emotional support.

① Never occurs ② Rarely occurs ③ Occasionally occurs ④ Frequently occurs ⑤ Always occurs

**g19**  48.I often feel disconnected from the people in my dormitory.

① Never occurs ② Rarely occurs ③ Occasionally occurs ④ Frequently occurs ⑤ Always occurs 　 　 　　　 　 　　　 　 　 　　 　 　　　　 　　 　　 　 　　　　 　　 　　 　 　 　 　**g20**  49.I perceive little meaning in living.

① Never occurs ② Rarely occurs ③ Occasionally occurs ④ Frequently occurs ⑤ Always occurs

**g21** 50. I often feel uneasy and suffer as a result.

① Never occurs ② Rarely occurs ③ Occasionally occurs ④ Frequently occurs ⑤ Always occurs

**g22** 51. Even when I set goals, I find it difficult to achieve them.

① Never occurs ② Rarely occurs ③ Occasionally occurs ④ Frequently occurs ⑤ Always occurs

**g23**  52. I feel that others do not like me.

① Never occurs ② Rarely occurs ③ Occasionally occurs ④ Frequently occurs ⑤ Always occurs

**g24**  53. When I need help, no one reaches out to assist me.

① Never occurs ② Rarely occurs ③ Occasionally occurs ④ Frequently occurs ⑤ Always occurs

**g25** 54. I frequently experience feelings of guilt.

① Never occurs ② Rarely occurs ③ Occasionally occurs ④ Frequently occurs ⑤ Always occurs

**g26** 55. Internally, I push myself to reach certain goals but worry more about my inability to succeed at them.

① Never occurs ② Rarely occurs ③ Occasionally occurs ④ Frequently occurs ⑤ Always occurs

**g27**  56. I feel incapable of meeting academic requirements.

① Never occurs ② Rarely occurs ③ Occasionally occurs ④ Frequently occurs ⑤ Always occurs

**g28** 57.I often believe that my performance is inadequate.

① Never occurs ② Rarely occurs ③ Occasionally occurs ④ Frequently occurs ⑤ Always occurs

**g29** 58.I feel lonely and perceive that no one is willing to accompany me.

① Never occurs ② Rarely occurs ③ Occasionally occurs ④ Frequently occurs ⑤ Always occurs

**g30**  59.I am dissatisfied with my current family relationships.

① Never occurs ② Rarely occurs ③ Occasionally occurs ④ Frequently occurs ⑤ Always occurs

**g31** 60.I feel a lack of presence within my family.

① Never occurs ② Rarely occurs ③ Occasionally occurs ④ Frequently occurs ⑤ Always occurs

Note: 1. grade: 1 = freshmen, 2 = sophomore, 3 = junior, 4 = senior. 2. Originating Place: 1 = Urban (county, city, province), 2 = Rural (township, town, village). 3. Whether you are the only child: 1 = yes, 2 = no. 4. D = Perceived Discrimination Questionnaire, E = Self-Esteem Scale, F = Sense of Life Meaning Questionnaire, G = college student psychological crisis screening scale
